# Identification of a Novel Homozygous Missense (c.443A>T:p.N148I) Mutation in *BBS2* in a Kashmiri Family with Bardet-Biedl Syndrome

**DOI:** 10.1155/2021/6626015

**Published:** 2021-02-23

**Authors:** Ghazanfar Ali, Sadia, Jia Nee Foo, Abdul Nasir, Chu-Hua Chang, Elaine GuoYan Chew, Zahid Latif, Zahid Azeem, Syeda Ain-ul-Batool, Syed Akif Raza Kazmi, Naheed Bashir Awan, Abdul Hameed Khan, Fazal-Ur- Rehman, Madiha Khalid, Abdul Wali, Samina Sarwar, Wasim Akhtar, Ansar Ahmed Abbasi, Rameez Nisar

**Affiliations:** ^1^Department of Biotechnology, University of Azad Jammu and Kashmir, P.O. Box 13100, Muzaffarabad, Pakistan; ^2^Lee Kong Chian School of Medicine, Nanyang Technological University Singapore, 11 Mandalay Road, Singapore 308232; ^3^Human Genetics, Genome Institute of Singapore, A∗STAR, 60 Biopolis Street, Singapore 138672; ^4^Molecular Science and Technology, Ajou University, Suwon, Republic of Korea; ^5^Department of Zoology, University of Azad Jammu and Kashmir, P.O. Box 13100, Muzaffarabad, Pakistan; ^6^Department of Biochemistry/Molecular Biology AJK Medical College, Muzaffarabad, Pakistan; ^7^Department of Chemistry Government College University Lahore, Pakistan; ^8^Department of Microbiology, Faculty of Life Sciences, University of Balochistan, Quetta, Pakistan; ^9^Department of Biotechnology, Women University of Azad Kashmir Bagh, 12500, Pakistan; ^10^Department of Biotechnology, Faculty of Life Sciences and Informatics, BUITEMS, 87100 Quetta, Pakistan; ^11^Department of Botany, University of Azad Jammu and Kashmir, Muzaffarabad, Pakistan; ^12^Department of Zoology, Mirpur University of Science and Technology (MUST), Mirpur AJK, Pakistan

## Abstract

**Background:**

Bardet-Biedl syndrome (BBS) is a rare autosomal recessive inherited disorder with distinctive clinical feature such as obesity, degeneration of retina, polydactyly, and renal abnormalities. The study was aimed at finding out the disease-causing variant/s in patients exhibiting clinical features of BBS.

**Methods:**

The identification of disease-causing variant was done by using whole exome sequencing on Illumina HiSeq 4000 platform involving the SeqCap EZ Exome v3 kit (Roche NimbleGen). The identified variant was further validated by Sanger sequencing.

**Results:**

WES revealed a novel homozygous missense mutation (NM_031885: c.443A>T:p.N148I) in exon 3 of the *BBS2* gene. Sanger sequencing confirmed this variant as homozygous in both affected subjects and heterozygous in obligate parents, demonstrating autosomal recessive inheritance pattern. To the best of our knowledge, this variant was not present in literature and all publically available databases. The candidate variant is predicted to be pathogenic by a set of in-silico softwares.

**Conclusion:**

Clinical and genetic spectrum of BBS and BBS-like disorders is not completely defined in the Pakistani as well as in Kashmiri population. Therefore, more comprehensive genetic studies are required to gain insights into genotype-phenotype associations to facilitate carrier screening and genetic counseling of families with such disorders.

## 1. Introduction

Bardet-Biedl syndrome (BBS; OMIM 209900) is a sporadic and genetically heterogeneous developmental syndrome. Primary clinical features of the syndrome include retinal degeneration, polydactyly, hypogonadism, severe kidney problems, and mental retardation along with obesity. Other secondary features associated with BBS are T2DM (type 2 diabetes mellitus), cardiovascular problems, hepatic diseases, hypothyroidism, and abnormal tooth development. The proposed clinical diagnosis of BBS is based on the presence of four major signs or three major and two minor ones. It is stated that BBS patients have insulin resistance that causes impairments in glucose metabolism and insulin sensitivity which subsequently leads to type 2 diabetes [1–4].

The prevalence of BBS in populations where the consanguineous marriages are low is relatively less, like in the population of Northern Europe and North America which ranges between 1/100,000 and 1/160,000, respectively, and in Tunisia which has been estimated at 1/156,000 [5]. However, it is more common in Kuwaiti Bedouins (1/13,500), the Faroe Islands (1/3,700), and Newfoundland (1/17,000) [6, 7]. These prevalence variations are the result of multiple reasons such as consanguinity, which is a social custom in nations such as Pakistan, Iran, Kuwait, and Middle East. The worldwide comprehensive epidemiological data is very less regarding BBS; even the Ashkenazi Jews, being apparently the most genetically studied founder population, have also not yet been exposed regarding BBS. So it is crucial to indicate that several of the document frequencies were not tailored to molecular investigations; consequently, the available number should be treated with care. Till now, very few cases of BBS have been documented in Asia, South America, Africa, and Eastern Europe, and an inclusive study remains to be done in these populations [8, 9].

To date, 21 BBS loci (*BBS1*-C8or f37/*BBS21*) (Table 1) have been implicated for BBS whose mutations would explain around 80.0% of affected patients [10, 11]. The study presented here was aimed at determining the clinical and genetic basis of BBS in two patients from a consanguineous Kashmiri family using whole exome screening, to broaden our understanding of spectrum, nature, and molecular basis of BBS in the Kashmiri population.

The state of Azad Jammu and Kashmir (AJ&K) is a bordering region between India and Pakistan; particularly, it is in the northeast of Pakistan and a relatively less developed region. There are no data available that could represent an inclusive picture of epidemiological studies of genetic diseases prevalent in the state. Therefore, further investigations are required to translate this knowledge for making proper clinical services to this kind of inherited disorders.

## 2. Methods

### 2.1. Ethics Statement

This study was permitted by the ethical committee/institutional review board of University of Azad Jammu and Kashmir Muzaffarabad. Written informed consent was obtained from the legal guardians of the affected subjects and also from the normal adults who participated in this study for the genetic testing and publication of photographs. After a detailed interview from the elders of the patients, the pedigree was built by using a method presented by Bennett et al. [12]. The pedigree of the family provided a significant indication of autosomal recessive inheritance pattern.

### 2.2. Genomic DNA Extraction and Whole Exome Sequencing

In order to investigate the disease-causing gene/s, peripheral blood samples were collected from both patients and clinically healthy subjects of the family. Genomic DNA extraction was done using phenol chloroform method [13]. For whole exome sequencing, we selected a clearly affected male (IV-3). Library enrichment for whole exome sequencing was conducted using SeqCap EZ Exome v3 kit (Roche NimbleGen). After that, enriched samples were sequenced by using an Illumina HiSeq 4000 (Illumina, San Diego, CA, USA). The average mean depth of the sequencing reads was kept up to 36x, and each read from the target region was covered ~94%.

### 2.3. Variant Calling

After the removal of adaptors and low-quality reads, the qualified raw fastq file was processed as per the recommendations of (GATK) “Best Practices for Germline SNP & Indel Discovery.” The clean sequences were mapped to UCSC Genome Browser hg19 by using Burrows–Wheeler Aligner (BWA-MEM v0.7.12), and subsequently, PCR duplicates were removed by using Picard v1.141. Finally, variant calling was done by using the GATK v2 Unified Genotyper.

### 2.4. Variant Annotation and Prioritization

All noncoding, synonymous variants with minor allele frequency (MAF) ≤ 0.01 in publicly available databases including Exome Aggregation Consortium (ExAC) (http://exac.broadinstitute.org/), 1000 Genomes, and Genome Aggregation Database (gnomAD) (https://gnomad.broadinstitute.org/) were excluded from the analysis. Only splice site, synonymous splice site variants, nonsense, nonsynonymous, indels that affect coding regions of gene were used for interpretation. Furthermore, the potential deleterious effect of the pathogenic variant on the structure and function of the protein was evaluated by using a set of in silico algorithms: SIFT (http://sift.jcvi.org/), PolyPhen2 (http://genetics.bwh.harvard.edu/pph2/), Mutation Taser (http://www.mutationtaster.org/), FATHMM (http://fathmm.biocompute.org.uk/index.html), Likelihood Ratio Test (LRT), and Condel [14].

### 2.5. Sanger Validation

The validation of expected candidate variant and extended segregation of candidate variants in all family members was done through Sanger sequencing.

### 2.6. 3D Modeling of the Protein

The amino acid sequence of *BBS2* protein was retrieved from the NCBI database (http://www.ncbi.nlm.nih.gov/), imported to pBlast search against Protein Data Bank (PDB). *Chaetomium thermophilum* (PDB: 6RXT) was used as a template for modeling of the three-dimensional structure of wild and mutant *BBS2* protein using I-Tasser software (Iterative Threading ASSEmbly Refinement) [15, 16]. Structural models were visualized by PyMOL (http://www.pymol.org). NCBI HomoloGene (http://www.ncbi. http://nlm.gov/Homologene/) was used to analyse the amino acid conservation among different orthologs.

## 3. Results

### 3.1. Clinical Report

**Figure 1:**
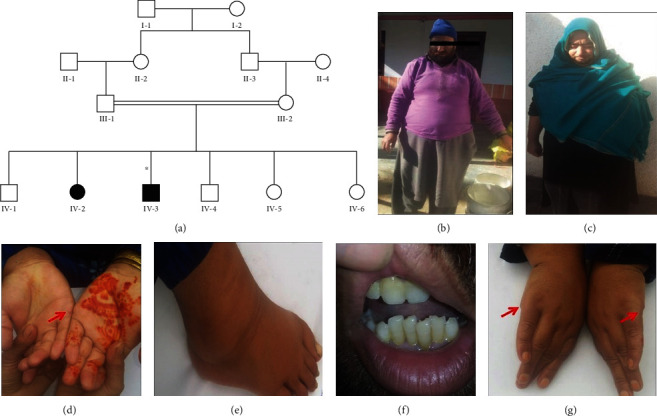
(a) A four-generation pedigree showing autosomal recessive mode of inheritance; solid squares and circles represent the affected male and female; however, unfilled symbols indicate the normal males and females. Asterisk (∗) indicates the individual used for WES. (b–g) Clinical phenotype in the individuals with BBS, confirmed by WES to have a novel missense mutation (c.443A>T:p.N148I) in exon 3 of the BBS2 gene, demonstrating the clinical manifestations: mild to moderate intellectual disability, visual impairment, postaxial polydactyly, and obesity. Red arrows in the figure indicate operated position.

In this study, a consanguineous Kashmiri family exhibiting clinical features of BBS was recruited from district Bagh Azad Jammu and Kashmir. The diagnosis was ascertained by following a previously established criteria for BBS [17]. The family comprises two affected subjects IV-2 and IV-3, and both were born from clinically normal parents who were first cousins by relation (Figure 1(a)). The patients exhibited obesity, renal problems, intellectual disability (ID), and postaxial polydactyly of hands and feet as a common phenotype. However, extra digits were operated in both patients at the time of the study (Figures 1(b)–1(g)). Patient IV-2 was reported with more severe ID than her brother (IV-3). These gross clinical investigations confirmed the diagnosis of BBS syndrome in both subjects. The affected male (IV-3) was also reported to have hypogonadism. Detailed clinical phenotypes are summarized in (Table 2).

All primary and secondary features investigated in both affected subjected are summarized in Table 2.

### 3.2. Molecular Findings

**Figure 2:**
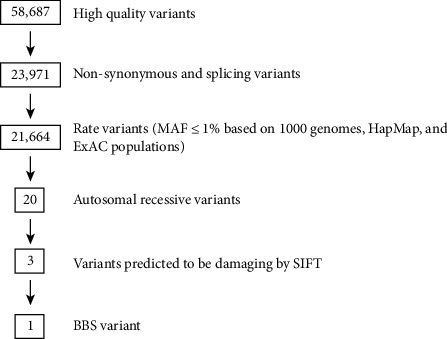
Flowchart for candidate variant filtering.

**Figure 3:**
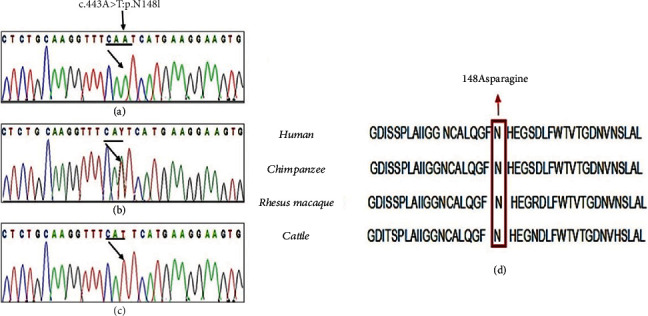
(a) Chromatogram of wild-type allele. (b) Chromatogram of heterozygous carrier parents. (c) Chromatogram of homozygous affected. (d) Multiple sequence alignment for human BBS2. Conservation of amino acid N among various ortholog species.

After exclusion of all the noncoding, nonsynonymous, and rare variants with MAF ≤ 0.01 across all assessed populations including 1000 Genomes, ExAC, and gnomAD, we got 21,664 variants in individual IV-3. All these filtration steps are mentioned in Figure 2. Then, based on the information collected from the previous literature (OMIM/PubMed) and pedigree illustration, we initially focused on homozygous variants; there were a total of 20 homozygous variants, and 3 of the variants were predicted to be damaging by SIFT and PolyPhen2 (Figure 2, Table 3). We then focused on the missense *BBS2* variant (NM_031885: c.443A>T:p.N148I) as the gene has been reported to be involved in BBS. The substitution of g16:56545099T>A occurs in exon 3 of the *BBS2* gene, leading to the N148I amino change. Sanger validation was done, and the variant was perfectly segregated among the individuals. Both patients were homozygous (Figure 3(c)), and their obligate parents were heterozygous (Figure 3(b)) for the mutation. However, the rest of the individuals were either heterozygous carrier or homozygous normal (Figure 3(a)).

This variant has not been reported before in the literature and also in any publicly available database. The variant was predicted to be potentially pathogenic by a set of other prediction algorithms as mentioned above.

## 4. Discussion

In this study, by using whole exome sequencing, we found a novel homozygous missense variant in the *BBS2* gene in a Kashmiri family. In addition, we also revealed WES combined with Sanger sequencing can be an effective strategy for molecular genetic diagnosis of BBS-like syndrome. BBS is a ciliopathy disorder that involves various body systems and mostly follows an autosomal recessive inheritance pattern; however, triallelic inheritance is also reported as a possible mechanism by some studies [18–20]. There are 21 genes that are associated with BBS so far; among those, *BBS1* and *BBS10* are mostly reported from North America and Europe, respectively, whereas *BBS2*, *BBS4*, *BBS5*, and *BBS12* are more prevalent in the Middle East and North Africa [11, 21, 22]. Based on previous and our current study, only eight BBS mutated genes (*BBS1* (OMIM #209901) [23], *BBS2* (OMIM #606151), *BBS3*/*ARL6* (OMIM #608845), *BBS5* (OMIM #603650) [24], *BBS8*/*TTC8* (OMIM #608132) [25, 26], *BBS9* (OMIM #615986) [9, 27], *BBS10* (OMIM #610148) [28–30], and *BBS12* (OMIM #610683)) [31] have been reported in the Pakistani population.

This study focuses on a consanguineous Kashmiri family having two affected individuals, one male (IV-3) and one female (IV-2) (Figure 1(a)). Both affected individuals exhibit established major symptoms of BBS, including obesity, retinitis pigmentosa, renal abnormalities, polydactyly, and learning disabilities. Other minor features and intrafamilial phenotypic variations were also noted among the affected individuals, like the affected female (IV-2) displayed more significant intellectual disability compared to her brother (IV-3). Bilateral postaxial polydactyly of both fingers and toes was identified upon clinical investigation in individual IV-2. However, the affected male (IV-3) has the only polydactyly of fingers. All these interfamilial and intrafamilial phenotypic variations are well described in BBS by previous studies as well [8, 32, 33].

**Figure 4:**
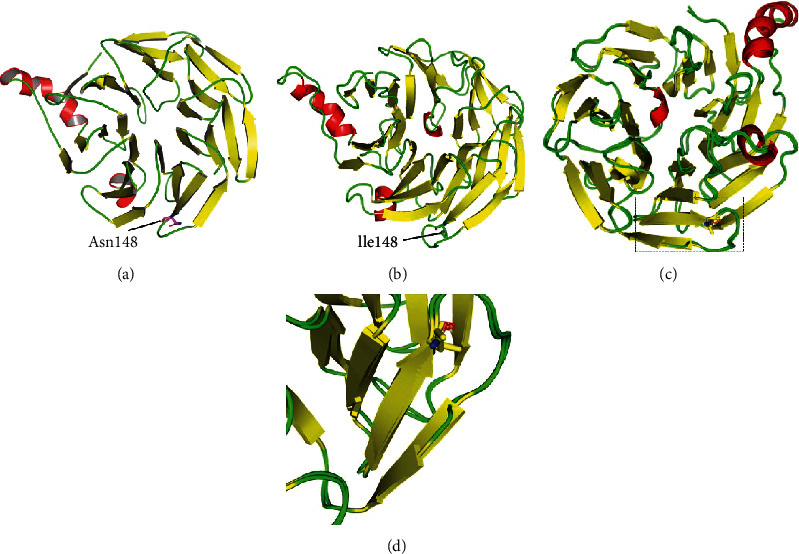
Comparison of wild-type BBS2 protein structure with its mutant form. 3D model of (a) wild- and (b) mutant-type BBS2 protein. (c) Superimposed structures of wild-type BBS2 protein and its mutant having mutation at position 148 showing the shortage of *α*-helix. (d) Close-up of the mutation.

WES revealed a novel homozygous missense variant (NM_031885: c.443A>T:p.N148I) in exon 3 of the *BBS2* gene. The position of the amino acid (N148) is highly conserved across different species (Figure 3(d)), and substitution is predicted to be damaging by in-silico analysis. This mutation was not present in any public database including dbSNP, 1000 Genomes, and ExAC. 3D protein modeling also indicated that the amide Asn residue in the mutant is replaced by Ile residue. The residue at position 148 in wild and mutant type is present at the loop region (Figures 4(a) and 4(b)). The mutation of polar amide side chain residue by nonpolar side chain caused a shortage of *β*-sheet in the nearby network as shown in Figure 4(d). As a consequence of this different, nature of amino acids and possibly different surface area may alter the function of the protein. The key function of the BBS genes is that they are involved in IFT (intraflagellar transport) and ciliary function. Seven genes (*BBS1*, *BBS2*, *BBS4*, *BBS5*, *BBS7*, *BBS8*, and *BBS9*) are involved in the formation of the BBSome complex, and *BBS6*, *BBS10*, and *BBS12* encode protein for the BBS chaperone complex that has a significant role in the maintenance and regulation of the BBSome [11, 34]. Mutations in *BBS2* genes are harmful because *BBS2* directly interacts with *BBS7* and *BBS9* to form the core complex that is vital for complete formation of the BBSome and ciliogenesis [1, 35].

Previous studies have reported that the *BBS2* gene is the third most frequent BBS gene to harbor mutational load (12.0%) after *BBS10* (20.0%) and *BBS1* (23.0%) [36, 37]. The cytogenetic location of the *BBS2* gene is on chromosome 16q21 and has 18 coding exons that encode 721 amino acid protein. There is a 45-amino acid coiled region in between exons 9 and 10, and this is flanked by peptide chain regions on both sides [38].

Although genetic studies have reported a total of 81 mutations including 49 missense/nonsense (HGMD, accessed on 30.12.2020) in the BB2 gene, only one mutation has been reported in the Pakistani population so far. This variant (Arg413Ter) was stated by Kordestani et al. in an annual meeting abstract in 2006 [39], and no detailed clinical information is provided in the abstract.

## 5. Conclusion

In conclusion, the present study reported a second novel missense c.443A>T:p.N148I mutation in the *BBS2* gene in a Kashmiri family from Pakistani origin. Furthermore, bioinformatics prediction and protein modeling confirmed the deleterious consequences of the identified variant on the *BBS2* protein structure. These results can be valuable in the future for more efficient analysis, genetic counseling, and prenatal diagnosis and also for population-specific screening.

## Figures and Tables

**Table 1:** List of candidate genes for autosomal recessive congenital BBS.

OMIM ID	Gene	Function	Cytogenetic position
209900	BBS1	BBSome protein	11q13
615981	BBS2	BBSome protein	16q21
600151	BBS3/ARL6	GTPase	3p12-p13
615982	BBS4	BBSome protein	15q22.3q23
615983	BBS5	BBSome protein	2q31
604896	BBS6/MKKS	Part of chaperonin complex	20p12
615984	BBS7	BBSome protein	4q27
608132	BBS8/TTC8	BBSome protein	14q32.1
607968	BBS9/B1	BBSome protein	7p14
615987	BBS10	Part of chaperonin complex	12q21.2
602290	BBS11/TRIM32	E3 ubiquitin ligase	9q31-q34.1
615989	BBS12	Part of chaperonin complex	4q27
609883	BBS13 MKS1	Centriole migration	17q23
610142	BBS14/CEP290/NPHP6	Basal body: RPGR interaction	12q21.3
613580	BBS15/WDPCP	Basal body: localization cx of septins and ciliogenesis	2p15
613524	BBS16 SDCCAG8	Basal body: interacts with OFD1	1q43-q44
606568	BBS17/LZTFL1	BBSome associated	3p21.31
613605	BBS18/BBIP1	BBSome	10q25.2
615870	BBS19/IFT27	Non-BBSome	22q12.3
608040	BBS20/IFT74	Non-BBSome	9p21.2
614477	BBS21/C8ORF37	Non-BBSome	8q22.1

**Table 2:** A brief diagnostic assessment of both patients.

	Case I (IV-2)	Case II (IV-3)
Sex/age (years)	F/28	M/26
Height (cm)/weight (kg)	146/71	150/73.5
*Major phenotypes*		
Retinitis pigmentosa	+	+
Obesity	+	+
Hypogonadism	+	+
Polydactyly (feet)	+	—
Polydactyly (hands)	+	+
Renal anomalies	+	+
Cognitive impairment	+	+
*Minor phenotypes*		
Hearing loss	—	—
Menstruation in female	Yes	—
Micro penis	—	Yes
Syndactyly	—	—
Diabetes mellitus	+	+
Heart problems	—	—
Hearing loss	—	—
Tooth pattern	Irregular	Irregular
Short neck, low nose bridge	+	+
Speech development	Delay	Delay
Growth condition	Normal	Normal
Behavioral expression	Hyperactive	Hyperactive
Eyesight	Weak	Weak
Facial morphology	Normal	Normal

**Table 3:** Potential deleterious nonsynonymous variants investigated through WES screening in affected individual (IV-3) from a Kashmiri family having clinical manifestations of BBS.

						Average allele frequency across global populations (Allele frequency in South Asians only)			Pathogenicity predictions		
	Gene	Ref	Alt	Amino acid substitution	Chr: position (Hg19)	1000 Genomes	gnomAD	ExAC	SIFT	PolyPhen2	OMIM disease
*Missense*	*BBS2*	T	A	N148I	Chr16:56545099	NA	NA	NA	Damaging	Benign	Bardet-Biedl syndrome
	*PKD2L1*	A	T	I520N	Chr10:102054392	NA	**0.000007953** (0.00006533)	0.00001647 (0.00006056)	Damaging	Probably damaging	NA
	*AOC2*	C	T	R279W	Chr17:40997478	NA	**0.0002333** (0.0001960)	0.0002 (0.0001)	Damaging	Possibly damaging	NA

## Data Availability

The data supporting the findings of this study will be available from the corresponding author upon request.
